# Catalytic Applications for Oxidized Carbon Materials: New Perspectives for the Special Issue in *Materials*

**DOI:** 10.3390/ma15176109

**Published:** 2022-09-02

**Authors:** Maria Rosaria Acocella

**Affiliations:** Department of Chemistry and Biology “A. Zambelli”, University of Salerno, Via Giovanni Paolo II, 84084 Fisciano, Salerno, Italy; macocella@unisa.it

This Special Issue from *Materials*, entitled “Catalytic Applications for Oxidized Carbon Materials”, aims to publish original papers on new scientific and applied research, providing great contributions to the understanding of oxidized carbon materials and related synthesis, characterization and applications.

The need for sustainable and metal-free catalysis has stimulated research on carbocatalysis to provide a new approach to the study of chemistry.

The possibility of efficiently conducting multiple reactions under solvent- and metal-free conditions, as well as the heterogeneous nature of the catalyst, has greatly stimulated and contributed to its growth.

It has been shown that graphite and graphene oxide are highly efficient and eco-friendly catalysts for many organic reactions [1,2], but other carbon materials need to be further investigated.

Furthermore, the ability of carbon materials to be functionalized represents an important tool for their application: changing functional groups can drive their catalytic activity differently, making them promising materials for a variety of applications in the industry.

Indeed, the ability of graphene oxide and oxidized carbon black to act as both filler and catalyst in nanocomposites was recently reported, mainly regarding thermoset resins [1,3] and rubber hardening [4].

The research interest of this Special Issue “Catalytic Applications for Oxidized Carbon Materials” includes, but is not limited, to the synthesis, characterization and catalytic application of different carbon materials (charcoal, carbon black, nanotubes, graphite), with particular attention to sustainability and reducing the environmental impact from the synthesis of the catalyst to its application.
